# Draft Genome Sequence of *Raoultella planticola*, Isolated from River Water

**DOI:** 10.1128/genomeA.01061-14

**Published:** 2014-10-16

**Authors:** Narayanan Jothikumar, Amy Kahler, Nancy Strockbine, Lori Gladney, Vincent R. Hill

**Affiliations:** a National Center for Emerging and Zoonotic Infectious Diseases, Centers for Disease Control and Prevention, Atlanta, Georgia, USA; b IHRC, Inc., Atlanta, Georgia, USA

## Abstract

We isolated *Raoultella planticola* from a river water sample, which was phenotypically indistinguishable from *Escherichia coli* on MI agar. The genome sequence of *R. planticola* was determined to gain information about its metabolic functions contributing to its false positive appearance of *E. coli* on MI agar. We report the first whole genome sequence of *Raoultella planticola.*

## GENOME ANNOUNCEMENT

U.S. drinking water regulations require that water samples be examined for fecal contamination using U.S. EPA-approved methods. Cultivation of membrane filtered water samples on MI agar has been used as a reliable method for detecting such contamination because of its ability to simultaneously detect and quantify both total coliforms and *Escherichia coli* specifically. MI agar is a selective and differential medium, containing cefsulodin to inhibit Gram-positive bacteria and noncoliform Gram-negative bacteria and indoxyl-β-D-glucuronide and 4-methylumbelliferyl-β-D–galactopyranoside for the presumptive identification of *E. coli* and other coliform bacteria, respectively (1). Although most coliform bacteria other than *E. coli* do not express β-glucuronidase activity, some strains of *Klebsiella* have been reported to express this enzyme and appear indistinguishable from *E. coli* on MI agar (2, 3). It is important to identify false-positive bacteria on MI agar to improve water quality monitoring techniques and risk assessments for the fecal contamination of water. A suspect blue colony of *E. coli* was isolated from a river water sample in 2012 on MI agar and was identified as *R. planticola* by *rpoB* gene sequencing (4). To understand the metabolic function of *R. planticola* strain CHB, whole genome sequencing was performed. The isolate was cultured in 10 mL Luria-Bertani broth overnight at 37°C. The overnight culture of 1 to 5×10^8^ CFU/mL was pelleted and resuspended in 350 µL PBS. The genomic DNA was extracted by mixing with an equal amount of 2×UNEX nucleic acid extraction buffer (Microbiologics, MN) as per manufacturer’s instructions followed by a final purification step using a polyvinylpolypyrrolidone (PVPP) spin column (Spin-IV-HRC columns, Zymo Research Corporation, Orange, CA).

The whole genome was sequenced using the Illumina MiSeq for paired-end 300×300 library preparation (MR DNA, Shallowater, TX). The genome sequence was assembled using DNAStar SeqMan NGen resulting in 18 contigs and a total length of 5.8 Mb. The genome had coverage of 422× (*N*_50_ of 561 Kb) and a G+C content of 55.4%. Annotation was performed using the RAST (Rapid Annotation using Subsytems Technology) server (5). The RAST server listed closest neighbors based on functional comparison of genome sequences, including *E. coli* AA86 (score 544), *E. coli* 96.0107 (score 284), *Klebsiella* sp. 1_1_55 (score 275), *Klebsiella pneumoniae* 342 (score 274), *Klebsiella oxytoca* 10 to 5,246 (score 250), and *Klebsiella variicola* At-22 (score 265). RAST predicted 5,462 coding sequences and 101 RNAs representing 572 subsystems. Several genes were identified that were associated with resistance to heavy metals (arsenic, cobalt, zinc, chromium, cadmium, mercury, and copper), as well as resistance to antibiotics [fluoroquinolones, fosfomycin, β-lactamase, multiple antibiotic resistance (mar) locus, the mdtABCD multidrug resistance cluster, and multidrug resistance efflux pumps]. Identification of antimicrobial resistance genes and carbohydrate metabolism of this bacterium will facilitate formulation of novel chromogenic/fluorogenic agar media to support exclusive growth and specific identification of *E. coli*.

### Nucleotide sequence accession numbers.

This whole-genome shotgun project has been deposited at DDBJ/EMBL/GenBank under the accession no. JPRG00000000. The version described in this paper is version JPRG01000000.
